# “Improving Access to Early Childhood Developmental Surveillance for Children from Culturally and Linguistically Diverse (CALD) Background”

**DOI:** 10.5334/ijic.4696

**Published:** 2020-04-22

**Authors:** Karen Edwards, Tania Rimes, Rebecca Smith, Ritin Fernandez, Lisa Stephenson, Jane Son, Vanessa Sarkozy, Deborah Perkins, Valsamma Eapen, Sue Woolfenden

**Affiliations:** 1 Counterpoint Consulting Pty Ltd, AU; 2 Children and Communities Program, South Eastern Sydney LHD, AU; 3 Child and Family Health Nursing, South Eastern Sydney LHD, Ramsgate, AU; 4 School of Nursing, Faculty of Science, Medicine and Health, University of Wollongong, Research and Education Building, Level 1, St George Hospital, Kogarah, AU; 5 Child and Family Interagency, South Eastern Sydney LHD, Caringbah, AU; 6 Developmental and Community, Kogarah Developmental Assessment Service, South Eastern Sydney LHD, AU; 7 Sydney Children’s Hospital, Randwick, AU; 8 Infant Child and Adolescent Psychiatry, University of New South Wales, AU; 9 Academic Unit of Child Psychiatry, South West Sydney (AUCS), AU; 10 Early Life Determinants of Health, Sydney Partnership for Health, Education, Research and Enterprise (SPHERE), AU; 11 Ingham Institute, AU; 12 BestSTART-SW (Systems Transformation and Research Translation – South West Sydney) Academic Unit, Randwick, University of New South Wales, AU; 13 ICAMHS, L1 MHC, Liverpool Hospital, AU; 14 Department of Community Child Health, Sydney Children’s Hospitals Network, AU; 15 School of Women and Children’s Health, UNSW Sydney CRICOS Provider Code 00098G, AU; 16 Discipline of Public Health, School of Public Health, the Faculty of Medicine and Health, University of Sydney, AU; 17 Conjoint lecturer, UNSW School of Women’s and Children’s Health, AU

**Keywords:** Child and Family Health Nurse, integrated care, developmental surveillance, child development, multicultural, CALD, migrant, refugee, non-government

## Abstract

**Introduction:**

Developmental vulnerabilities in pre-school aged children from culturally and linguistically diverse (CALD) backgrounds with low English proficiency are less likely to be identified through universal developmental surveillance. Barriers include low parental health literacy and low rates of attendance to mainstream child and family health services. Late detection of developmental vulnerabilities can have lifelong impacts on life trajectory.

**Method:**

Integrated outreach early childhood developmental surveillance was trialled in South East Sydney by local health services with non-government organisations (NGO) delivering early childhood education and support. NGO staff were trained in Parents Evaluation of Developmental Status (PEDS), a validated developmental screening tool to explore parental/carer and provider concerns [1]. Families with children identified with developmental concerns by NGO staff were referred to co-located or visiting Child and Family Health Nurses (CFHN), community child health, speech pathology or developmental services for developmental screening, assessment and/or care planning.

**Results:**

Integrated health and NGO services improved access to developmental surveillance for CALD families in a non-threatening environment enabled by co-locating CFHN, or through visits by paediatric medical/speech pathology staff to participating playgroups.

**Conclusions and discussion:**

Integration supported vulnerable families from CALD backgrounds to access developmental surveillance through child and family health services but required flexibility and adjustments by all involved.

## Introduction

### The problem

Children from culturally and linguistically diverse (CALD) backgrounds, who are not proficient in English at school entry (for a variety of reasons) are 1.5 times more likely to have developmental vulnerabilities across the spectrum of concerns [2,3,4]. Children from CALD backgrounds face additional challenges in accessing early identification and intervention for developmental delays due to a mix of cultural beliefs, parental knowledge and characteristics of the early childhood service system [5,6,7,8]. The life course for such children is compromised from this early beginning and, if not addressed, can have an adverse impact on their entire life trajectory [6]. Therefore, it is important to identify individual children with developmental vulnerabilities as early as possible in the preschool years so that they can be referred for early intervention.

In NSW, child and family health services offer universal developmental surveillance of children 0–4 years through a mixture of home visiting and clinic-based services. Child development checks are carried out according to a schedule contained in the child’s Personal Health Record (PHR or “Blue Book”). Although intended as a universal service, this service does not necessarily reach all families. Lower economic status, parental lack of English language proficiency, belonging to certain ethnic groups and lower levels of maternal education are all associated with less attendance at early childhood health services [7]. A 2014 national survey of CFHN reported there was generally a rapid drop-off in contact with CFHN for developmental checks after 6 months [8]. In NSW a rapid review undertaken for the Sax Institute found NSW experienced a similar drop-off [9].

Early childhood education and support services present another opportunity for engaging with families who might not otherwise attend a Child and Family Health Centre. Families with vulnerabilities may be identified through the social care system, or by their community connections (especially in CALD communities) and be provided with the opportunity to attend supported playgroups, family support services or childcare.

This represents an opportunity for child and family health services to reach vulnerable children through supported playgroups and other non-government early childhood services. Under this model, while technically all children have access to a base level of universal support through centre-based services, children from families with additional vulnerabilities have access to additional tailored support to address specific needs in a more accessible location. The purpose of this case study is to describe an outreach developmental surveillance model that leveraged off the work of child and family health services that support children aged from six weeks to four years of age, from CALD backgrounds.

### The setting

Rockdale and Botany are two suburbs in South Eastern Sydney, noted for high CALD populations and high socioeconomic disadvantage. In the 2016 Census Rockdale reported a population of 109,404, with 63.6% reporting to be born overseas. Botany reported a population of 10,817, with 36.8% reporting to be born overseas [10].

In Rockdale, 80.1% of all children living in the local government area had attended a pre-school or kindergarten, and 44.9% had attended playgroup and/or day care and/or family day care before starting school. In Botany 76.3% of all children had attended pre-school or kindergarten and 50.7% had attended playgroup and/or day care before starting school [11]. Research conducted in 2013/2014 with the families from CALD backgrounds and non-government family support providers in the Botany and Randwick areas of South Eastern Sydney identified the following service provider barriers to universal developmental surveillance in preschool children [12]: a lack of understanding of the role of CFHN; child and family non-government (NGO) services who had no specific training in early detection of developmental and behavioural vulnerabilities in preschool children; challenges in how service providers can communicate their concerns about a child’s development with the child’s family; and a lack of clarity regarding referral pathways from initial concern to further assessment.

### The solution

**Figure 1:**
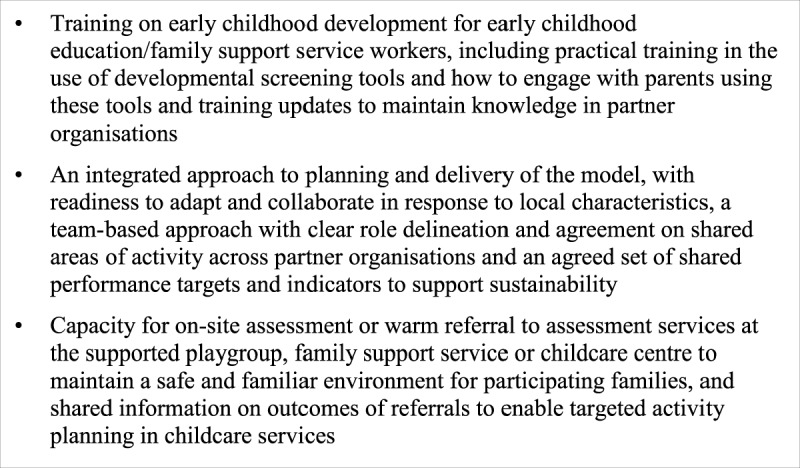
Core Enablers to Integrated Model of Early Childhood Developmental Surveillance.

As a result of the findings from the above research, models to improve early childhood surveillance for children from vulnerable families (especially families from CALD backgrounds) were developed and trialled at Botany and Rockdale, in the context of NGO early childhood education/family support services. Figure 1 below shows the core enablers of the models introduced to address the barriers identified.

**Figure 2:**
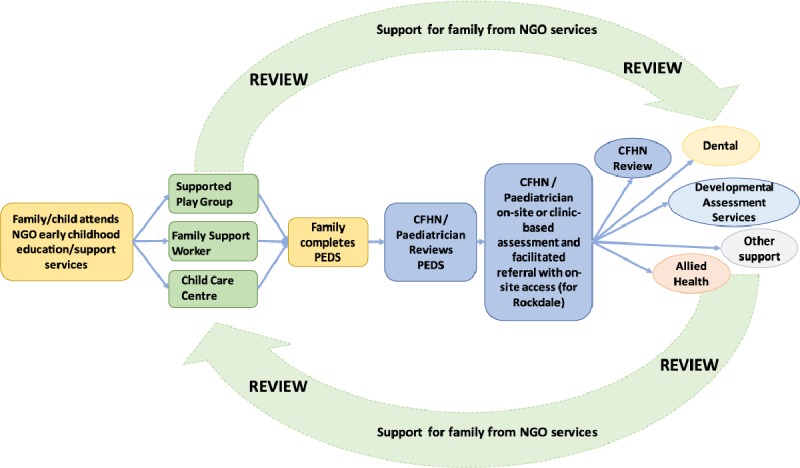
Rockdale and Botany Integrated Model Flow.

The integrated approach involved a flow of activities to identify parents with vulnerabilities and guide them through the early childhood developmental surveillance pathway. The model below captures the flow of the projects (Figure 2).

## The components

### Training

**Figure 3:**
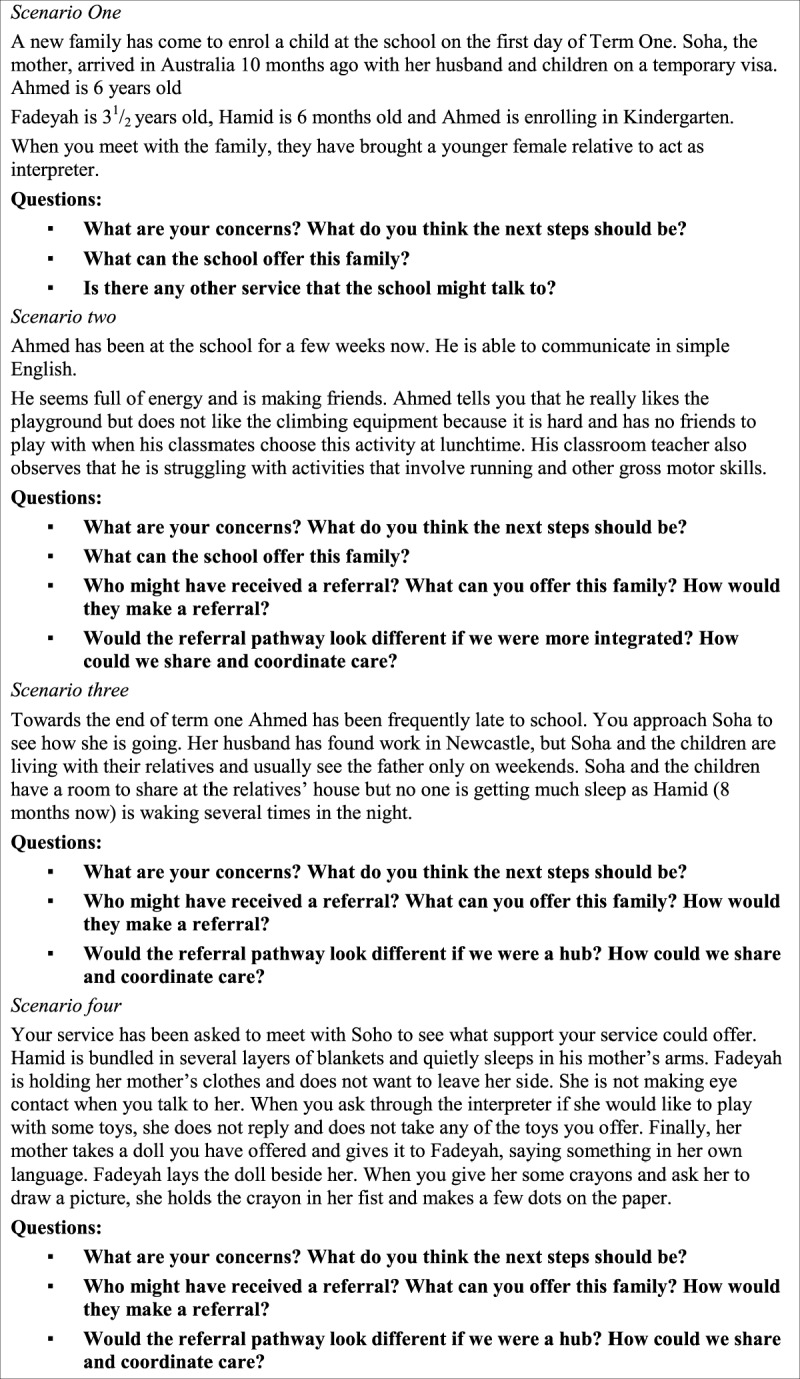
Case study scenario used in training.

Training was provided to staff working in participating non-government early childhood/family support services in half day blocks. The content of the training included: early childhood development; developmental milestones; training on the “Blue Book”, the parent held NSW personal health record for infants and children up to age five; administration of the Parents Evaluation of Developmental Status (PEDS), a universal developmental surveillance tool; and child protection. Twenty-two participants across four sites attended the training. The design and content of the training was aimed at childcare workers, family support workers and supported playgroup staff. Case scenarios allowed exploration of likely issues and possible responses. A sample case study that was used in the training is shown in Figure 3.

Pre and post-training surveys were administered to attendees to assess changes in knowledge as a result of the training. The questionnaire was completed by study participants prior to completion of the training and re-administered two months after the training (n = 16). Attendees reported acquiring additional knowledge and or skills in relation to developmental surveillance. After the training, all participating staff reported referring to child and family health services for developmental concerns. For participants with early childhood education qualifications, the information on child development and milestones was seen as reinforcing their existing knowledge. The use of case studies as a means of cementing knowledge and providing practical experience, was appreciated by participants. For those not familiar with the Personal Health Record (the Blue Book) and the PEDS, this aspect of the training helped increase their understanding of these tools and how they could use them to talk with parents about developmental concerns.

### Delivery

Service delivery characteristics for the two models are summarised in Table 1.

**Table 1:** Characteristics of Botany and Rockdale Models.

Characteristics	Botany Model	Rockdale Model

Partners	Non-government local providers of multicultural supported playgroups and parenting groups	Non-government early childhood service provider comprised a Children’s Centre, Supported Playgroup and Family Worker services
Site	Various depending on location of playgroups	Single facility owned by Non-government service provider
Health providers	Community Child Health Doctor and/or Speech Pathologist from the Sydney Children’s Hospital Network (SCHN). In 2017, a Child and Family Health Nurse commenced visiting the playgroups on a rotating basis.	A Child and Family Health Nurse based on-site two days per week.
Assessment	Assessments made on-site or nearby, and referrals made to a Developmental Assessment Clinic if required	Developmental checks made on site with referrals to Developmental Assessment Clinic if required. Developmental Assessment Service held monthly clinics on site (2 appointments).

Staff in the non-government early childhood/family support services received feedback and advice from health providers on how to support children and parents where developmental issues were identified and worked with families to help them to undertake recommended actions to assist their children. The regular contact with early childhood health professionals was viewed by the health and non-government services as a strong enabler in encouraging parents to follow up referrals and remain engaged.

Of a randomly selected sample dataset of children seen by the CFHN at the Rockdale project from November 2016 to June 2018, 79% (n = 54) were children of mothers whose country of birth was not Australia. The top five countries of birth of these mothers were Nepal, India, Vietnam and Bangladesh. Mothers born in Nepal or Bangladesh were least likely to report speaking English at home, with one out of eight Nepalese mothers recorded as speaking English at home and nil out of five Bangladeshi mothers. The Rockdale NGO employs a Nepali-speaking manager, who was one of the two lead staff engaged with the project.

The key areas of concern identified in children at Rockdale were behaviour, expressive language and articulation, gross motor skills, social/emotional concerns, fine motor skills and global/cognitive concerns. Referrals were made to a range of services including speech therapy services, developmental assessment services, oral health, psychological services and other NGOs.

Focus groups with families in Rockdale and Botany revealed that very few of the participants delineated between the CFHN service available through the supported playgroup, and the supported playgroup itself. Questions regarding CFHN were answered in terms of the overall experience with the supported playgroup attended. Where parents in focus groups differentiated between playgroup and CFHN, they were able to describe the support they received from CFHN as including advice about eating and sleeping, establishing routines, dental care and tips for playing with children.

Co-location was recognised as a significant enabling factor in parents attending initial assessments and completing further developmental assessments. The on-site visits from the Developmental Assessment Service were identified as an enabler for some families to take the next step to additional assessment and support. Supported playgroup providers in Botany noted the value of parents meeting the Community Child Health Doctor and CFHN through the playgroup and becoming familiar with them in that environment. Both projects have evolved as circumstances have changed, through open discussion and problem-solving across the participating services.

## Discussion

Traditionally, provision of early childhood developmental surveillance (after the initial 4-week check) has been through attendance by parents/carers at a Child and Family Health Centre. The results of the Botany and Rockdale projects indicated that there must also be other ways of engaging other families. Health and non-government services reported that this model was particularly important to identify and engage families with additional vulnerabilities who might otherwise ‘slip through the net” of a universal child health surveillance system.

There is limited evidence regarding this type of integrated approach, however in Canada and in Australia, integrated approaches to increasing developmental surveillance in vulnerable populations have been trialled. In Canada the Social Paediatrics Initiative implemented a model of care that worked closely with local disadvantaged communities and non-government organisations to increase access to early childhood assessment, primary health care services and specialist services. This was a collaborative approach between health care providers, community members and community non-government organisations working with children and families and included ‘in-place’ assessment at early childhood facilities where this appeared the best way to improve access. The initiative reported improved engagement of vulnerable families with the early childhood health system and improved access for children and families to specialist support [13].

In the state of Victoria, in Australia, The Wodonga Early Years’ Service Coordination Framework tested the Parents’ Evaluation of Developmental Status (PEDS) with childcare workers, pre-school teachers and primary school teachers, as well as child and maternal health services. The PEDS was reportedly easy to use across these different service providers and was also well-received by parents. High risk children were identified through the use of the PEDS in early childhood education environments and referrals to specialist services were made. There was differentiation between high, medium and low risk children with subsequent capacity for early childhood service/education providers and child and maternal health services to appropriately refer [14].

### Lessons learned

There were a number of key lessons learnt from the Rockdale and Botany projects. Because the projects were a new way of working, services had to adjust and reconsider their traditional processes. Early childhood education/family support service providers also noted that the results of the projects had shaped the way they worked, for example with transition to school.

Participants noted the time it took to establish the projects, including building relationships between participating organisations, identifying required resources and infrastructure, and making the necessary investments. In Rockdale the health service established a part-time co-ordination role to help set up training and establish processes and protocols, and to facilitate the planning and early implementation of the project. In Botany, this work was done within existing workloads, which was challenging.

During the set up and early implementation there was a degree of experimentation. As the projects bedded down, early experiences shaped the final model, for example, the most efficient ways to book appointments and manage data. In Rockdale, the co-ordination role became part of core business as the project bedded down, with ongoing co-ordination activities becoming part of the role of a senior program coordinator.

There was recognition by health service managers and health professionals that this model of care required resources which might be additional to those expended in a more traditional model. Examples included access to cars, use of mobile equipment that could be easily transported and mobile access to data systems. Early childhood/family support service providers also reported that setting this model up meant additional time and use of their limited resources. They spoke of their commitment to making the extra time in order to meet the needs of their community. It is important that this commitment is recognised (as it has been in these projects) and acknowledged, without being taken for granted.

The models differed in some aspects of staffing and sites to respond to local context and proactively helped build a model that worked for the partners and the families. The results of this approach were positive in terms of proportions of children screened and potential concerns identified. The message here is clear. Keep the core elements, but be ready to adapt and flex to the local environment and context. Early childhood/family support service providers and health professionals and managers noted the importance of recognising and respecting the different cultures, and the different priorities of the partner organisations. For example, CFHN might require specific clinical spaces in which to undertake developmental checks, but this could have been an impost on a well-used community building with space restrictions.

A key success of these initiatives has been increasing access for families to services and improving the reach of services to families. For example, recognising the amount of oral health issues in children at the Rockdale early childhood education/family support services, the health service arranged for the Oral Health Service to visit the service. Staff at the service had noted a lower proportion of families accessing dental health services in Rockdale till then. Several children with high oral health needs were identified and dental hygiene became a key topic of discussion. This was reflected in the feedback from one of the parent focus groups.

One of the outcomes from the project in Rockdale has been the involvement of the Rockdale Primary School and the establishment of the Rockdale Hub. A key driver for this initiative was the issue of children with additional support needs starting school without the school being aware beforehand of their developmental needs. This meant that the school had not made arrangements to support these children at school from their date of commencement. The Principal is now encouraging parents to enrol their children earlier in the year prior to school entry and is undertaking individual interviews with families prior to school entry to discuss developmental issues and school readiness.

This has expanded the team at Rockdale to include the school and other community-based services supporting children and families.

## Conclusion

This case study describes integrated models of care where health works in partnership with non-government early childhood services to increase access for vulnerable families, especially those from culturally and linguistically diverse backgrounds, to early childhood developmental surveillance.

Often health services deliver programs or outreach services to different settings without truly integrating into that setting. The level of service integration achieved by the projects has been effective in building capacity across sectors and achieving systems change.

Successful implementation of this integrated approach required: an existing ‘bank’ of good will between participating organisations; identification of a shared priority (developmental surveillance of vulnerable children) between participating organisations; a clearly defined target population; mutual respect for the roles and business models of participating organisations; openness and honesty in dealings between participating organisations; shared planning and ongoing monitoring of the projects; flexibility and adaptation in the face of identified challenges or barriers; and investment and commitment of time and resources.

Two critical enablers were the readiness of the health services involved to step out of their traditional clinic-based service models into highly flexible community environments, and equally, the willingness of NGOs to adapt and make space for health services that have not previously been accommodated into their business and their facilities. The degree of trust and ongoing negotiation required for success cannot be underestimated.

This study reports on a limited sample in two metropolitan locations. Replication in another location with a population living with adversity and marginalization is needed to further test the model. Ideally this would include a randomised controlled trial. The identified challenges and enablers for the successful implementation of these integrated models of practice provide a series of lessons to be applied if they are to be scaled up and replicated.
